# Construction enthusiasts versus demolition giants: Insights from building footprint data in England

**DOI:** 10.1177/23998083251317573

**Published:** 2025-01-30

**Authors:** Xinyi Yuan, Ziqi Li, Ana Basiri, Mingshu Wang

**Affiliations:** 1 School of Geographical and Earth Sciences, 3526 University of Glasgow, Glasgow, UK; 2 Department of Geography, 7823 Florida State University, Tallahassee, FL, USA

**Keywords:** Building footprint data, urban development, construction, demolition, England

## Abstract

This study uses building footprint data from the Ordnance Survey MasterMap to analyze construction and demolition activities across England from 2017 to 2023. By comparing the Topographic Object Identifiers (TOIDs) of each building between years, we identified newly constructed and demolished buildings, quantified changes, and used the bivariate color maps to visualize spatial patterns across England and within its five major cities. The study highlights the effectiveness of building footprint data in providing insights into urban changes and development trajectories, which are vital for urban planners and policymakers to understand dynamic urban processes and inform decision-making toward sustainable urban development.

## Main text

Buildings are core elements of the urban environment, and construction and demolition activities are fundamental to urban development (Alexander, 1977; Lynch, 1960). These processes reshape cities, influence socioeconomic conditions, and determine future urban trajectories (Balaban, 2012; Perez et al., 2021; Wu, 2014; Yin et al., 2023). Understanding the dynamics of construction and demolition is essential for urban planning and policymaking.

Traditional datasets are often inadequate for individual building-level analysis. Satellite imagery can monitor urban expansion but struggles to identify subtle changes in densely built areas. Street view imagery can detect building changes but is challenging to quantify systematically at a large scale. Compared to these datasets, building footprint data—representing building outlines—are finely sampled and frequently updated, providing comprehensive coverage. This makes building footprint data promising for assessing urban construction and demolition at fine scales.

Our study used building footprint data from Digimap Ordnance Survey (OS) MasterMap to quantify building changes and visualize construction and demolition patterns in England from 2017 to 2023. The OS MasterMap provides unique lifecycle Topographic Object Identifiers (TOIDs) for each building footprint, enabling effective change tracking. By comparing TOIDs between the 2017 and 2023 datasets, we identified newly constructed buildings (TOIDs absent in 2017 but present in 2023) and demolished buildings (TOIDs present in 2017 but absent in 2023). To explore the relationship between demolition and new construction patterns across England, we calculated the areas of these changes and used a bivariate color map to visualize the per capita new construction and per capita demolition areas at the Local Authority District (LAD) level across England and the Lower Layer Super Output Area (LSOA) level in its five major cities (i.e., Greater London, Birmingham, Manchester, Liverpool, and Leeds). Population data were sourced from the Census 2021. The color palette was selected from Brewer’s (1994) “Color Use Guidelines for Mapping.” Both variables were classified into three categories (low, moderate, and high) based on the 1/3 and 2/3 quantile breaks, resulting in nine color combinations to distinguish areas with varying levels of construction, demolition, and their overlap.

**Figure 1.**
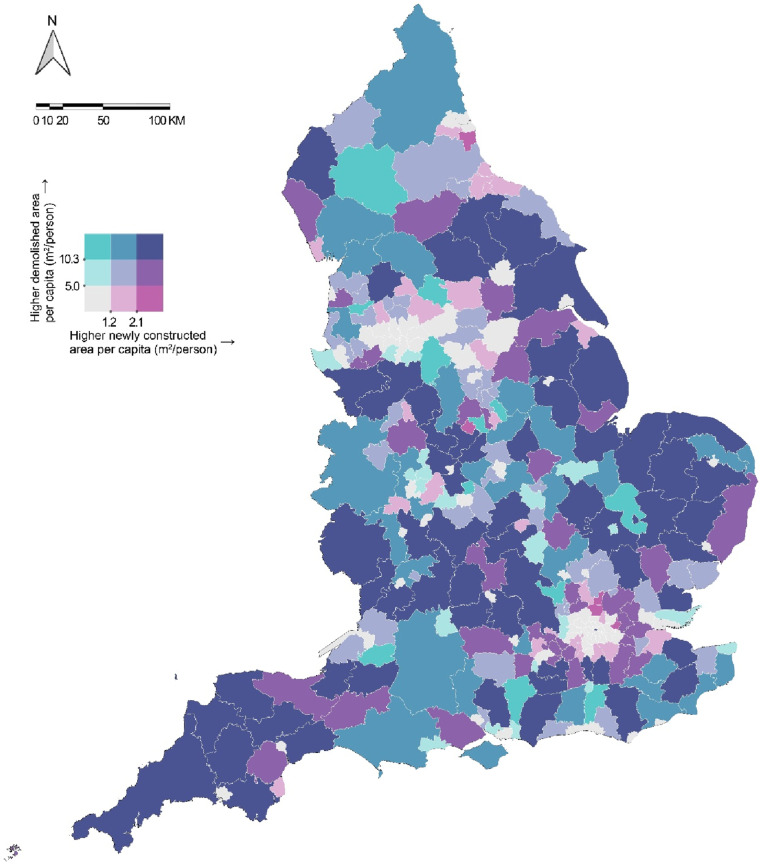
Construction and demolition patterns in England by Local Authority District (LAD), 2017–2023.

**Figure 2.**
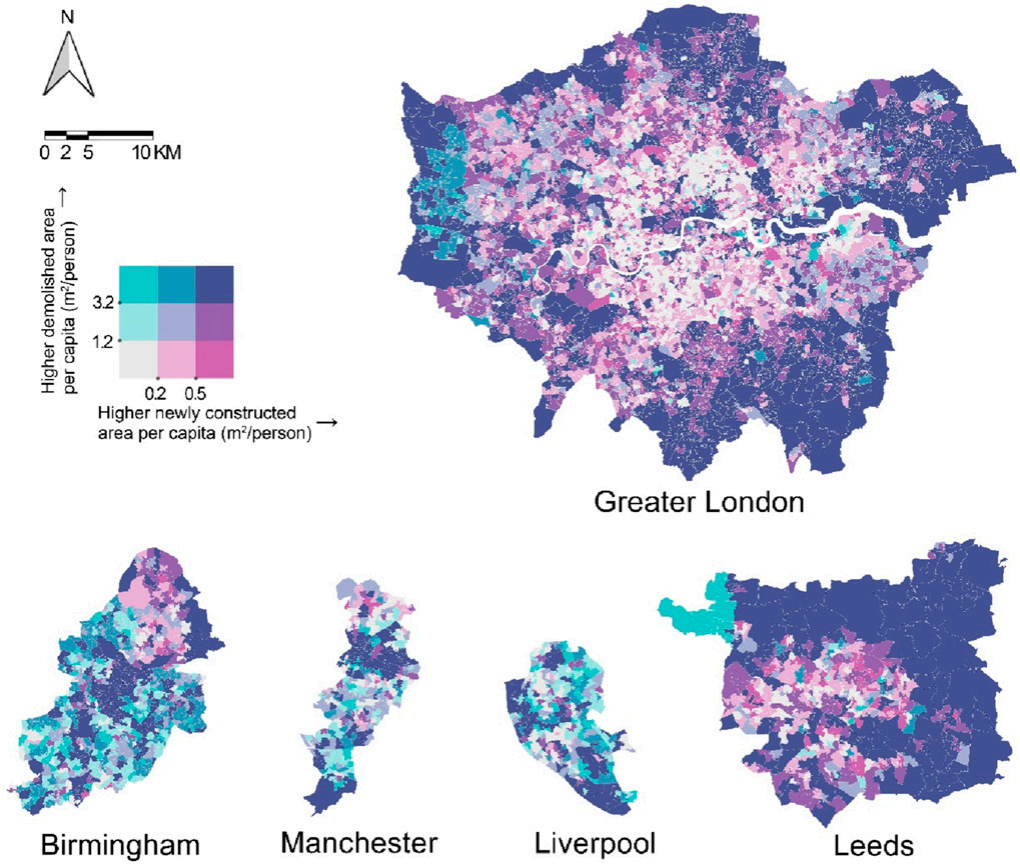
Construction and demolition patterns of England’s five major cities by Lower Layer Super Output Area (LSOA) and Local Authority District (LAD), 2017–2023.

Figure 1 illustrates construction and demolition patterns in England at the LAD level from 2017 to 2023. Metropolitan cores, such as London and Manchester, exhibit relatively low construction and demolition intensities, while their surrounding and more distant areas show moderate development and redevelopment activities. In contrast, some LADs characterized by countryside and smaller towns demonstrate higher construction intensity. Figure 2 focuses on five major cities in England to better understand the spatial patterns within cities, revealing the differences in construction and demolition activities and urban development trajectories over the past 5 years. In Greater London, the central areas, except the City of London, show low levels of demolition and construction, while the outer areas exhibit high levels overall, reflecting a development pattern focused on outward expansion. In contrast, Birmingham and Manchester show an opposite pattern, with high activity concentrated in the city center and surrounding areas, indicating extensive redevelopment of central areas, while the outer regions exhibit limited activity. Liverpool’s development patterns appear notably dispersed, with high demolition and construction activity occurring in the city center, along the River Mersey, and across various other urban districts. Leeds has mixed activity levels in the city center and surrounding areas, with high-intensity activity in the northern and eastern regions.

In conclusion, our study demonstrates the utility of building footprint data for tracking construction and demolition activities across large urban areas. The analysis reveals England’s construction and demolition trends over 5 years and highlights distinct city patterns. These insights are crucial for urban planners and policymakers, as they highlight areas of dynamic change and can guide decision-making related to sustainable urban growth and resource allocation.

## Data Availability

The building footprint data are available via Digimap upon request at https://digimap.edina.ac.uk/.
